# Reply to Letter to the Editor : Is HPV-18 present in human breast cancer cell lines

**DOI:** 10.1038/sj.bjc.6605672

**Published:** 2010-04-20

**Authors:** B Heng, W K Glenn, J H K Lee, X V Tan, J S Lawson, N J Whitaker

**Affiliations:** 1 School of Biotechnology and Biomolecular Sciences, University of New South Wales, Sydney, New South Wales, Australia

**Sir**,

In response to the lack of detection of HPV in MDA-MB-175VII and SK-Br-3 cell lines, we note that the authors have not followed the methods as set out (Heng *et al*, 2009). Critically, Peran *et al* (2010) relied on single rounds of standard and reverse transcription (RT)–PCR. In our report, we specifically indicate the use of nested PCR (using the MY11/MY9 followed by GP5/GP6 combination for amplification of the L1 region of HPV). The use of nested primers was necessary to increase the sensitivity without compromising the specificity of the standard PCR.

The requirement for the increased sensitivity for detection of HPV in the breast cancer cell lines may indicate less than one copy of HPV per cell, unlike the situation in cervical cancer cell lines such as HeLa. Ever since reporting the detection of HPV-18 in the MDA-MB-175VII and SK-Br-3 breast cancer cell lines, we have endeavoured to further characterise the HPV genome present in these cell lines. This analysis is by no means complete, but we present our relevant preliminary findings here.

The breast cancer cell lines MDA-MB-175VII and SK-Br-3, and all cell lines in our laboratory, have been subjected to ‘DNA fingerprint’ analysis by SNP PCR (summarised in Table 1). All cell lines have different DNA fingerprints, demonstrating that these cell lines are not contaminated. In addition, we have confirmed that all cell lines are free from Mycoplasma infection (example shown in Figure 1).

We reported the amplification of the L1 region by PCR using nested primers (MY/GP) from DNA isolated from both MDA-MB-175VII and SK-Br-3 cell lines (Heng *et al*, 2009). This product was also sequenced to confirm the identity of HPV-18. Here we report that we have successfully amplified E6 by standard PCR from MDA-MB-175VII DNA and by RT–PCR from RNA, indicating the presence of E6 and the expression of E6 in these cells (Figure 2). The RT–PCR bands from Figure 2 were isolated, purified and subjected to sequence analysis. As shown in Figure 3, the sequence of this E6 region was identical in HeLa and MDA-MB-175VII cell lines. This is not unexpected given that this region is functional and conserved.

Because of the difficulty in amplifying HPV in these breast cancer cells and because these cell lines have been available and passaged in many different laboratories over many years and may have changed significantly over time, we subcloned (by limiting the dilution) the MDA-MB-175VII cell line. Here we show that all six subclones of MDA-MB-175VII have the same DNA fingerprint as the parental MDA-MB-175VII cell line (Table 1), as expected. Interestingly, all the clones contained a mixture of fusiform and epithelial-shaped cells, indicating that the single clones vary or alternate in phenotype and that these two forms (need to?) coexist for the continued proliferation of the cell line.

Only three of six MDA-MB-175VII clones isolated apparently contained HPV as indicated by the successful amplification of the L1 region by nested PCR and by *in situ* PCR as per Heng *et al* (2009). Interestingly, the HPV-containing clones grew faster than the clones without amplifiable HPV.

We have confirmed the presence of HPV in the nuclei of both MDA-MB-175VII and SK-Br-3 cell lines by *in situ* PCR subsequent to the report in Heng *et al* (2009).

By using a series of primers in the L1 region, it appears that the L1 sequence does not extend 5′ far beyond GP5 in the MBA-MB-175VII cell line. The region 3′ of the L1 amplicon was extended at least as far as 7244 bp. This suggests that the HPV is fragmented and/or incomplete and may indicate ‘hit-and-run’ oncogenesis as described for HPV in Syrian hamster embryo cells (Iwasaka *et al*, 1992) and for human mesothelial cells (De Silva *et al*, 1994). We are continuing our analysis of the HPV sequence in MBA-MB-175VII and SK-Br-3 cell lines, as well as in two other HPV-containing breast cancer cell lines that are yet to be described and characterised.

We conclude that the HPV sequences are present as reported (Heng *et al*, 2009). We suggest that the difficulty in detection is possibly due to (i) the loss and/or fragmentation of the HPV (suggesting hit-and-run transformation), and/or (ii) instability or variability of the HPV-containing cell lines.

## Figures and Tables

**Figure 1:**
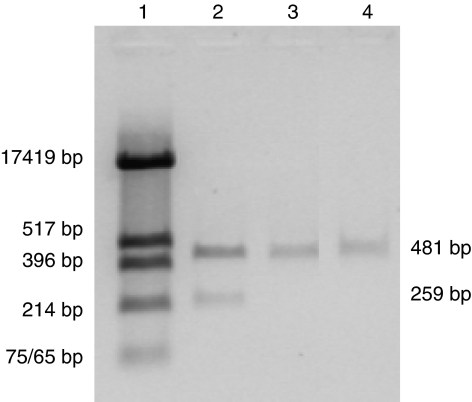
Mycoplasma testing of breast cancer cell lines. DNA isolated from the cell line supernatant was amplified with the control and mycoplasma-specific primers (Sigma LookOut Mycoplasma PCR Detection Kit (MP0035)); products were resolved by electrophoresis in a 1.2% agarose gel. The presence of the 259-bp band indicates the presence of Mycoplasma sequences, while the 481-bp band indicates that the PCR reaction worked. Lane 1: pUc-*HinfI* size marker, lane 2: Mycoplasma-positive control, lane 3: negative control, lane 4: SK-Br-3.

**Figure 2:**
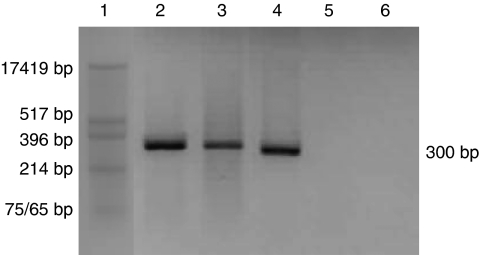
E6-reverse transcription–PCR. Lane 1: Puc/Hinf marker, lanes 2 and 3: cDNA from HeLa and MDA-MB-175VII, respectively, lane 4: HeLa genomic DNA (postive control), lane 5: RT–PCR reaction without the RT enzyme (RT control), lane 6: water (PCR-negative control). HeLa cDNA and genomic DNA, and MDA-MB-175 VII cDNA, yield a positive band of 300 bp, indicating expression of E6 mRNA in both HeLa and MDA-MB-175VII cell lines. Primer sequences: E6 forward primer, 5′-CGGCGACCCTACAAGCTAC-3′, E6 reverse primer, 5′-GCACCGCAGGCACCTTAT-3′.

**Figure 3:**
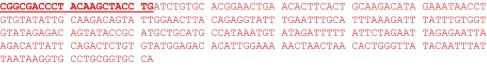
Sequencing of the E6 RT–PCR product in breast cancer cell line MDA-MB-175VII. The products from the RT–PCR were purified and sequenced. The resultant sequence is shown and is identical to the E6 RT–PCR sequence obtained from the HeLa E6 RT–PCR analysis. The underlined sequence is the forward E6 PCR primer.

**Table 1:** DNA fingerprinting of HPV-containing breast cancer cell lines

**Cell line/SNP marker^a^**	**D18S63**	**D18S452**	**D6S1035**
Negative	Negative	Negative	Negative
HeLa	1 (270)	1 (151)	2 (145, 151)
SiHa	2 (270, 284)	1 (151)	2 (143, 156)
C33A	2 (272, 290)	2 (161, 163)	2 (139, 151)
SK-Br-3	2 (282, 286)	1 (155)	2 (143, 151)
MDA-MB-175VII	2 (270, 286)	1 (155)	2 (150, 154)
175 Clone 1	2 (270, 286)	1 (155)	2 (150, 154)
175 Clone 2	2 (270, 286)	1 (155)	2 (150, 154)
175 Clone 3	2 (270, 286)	1 (155)	2 (150, 154)
175 Clone 4	2 (270, 286)	1 (155)	2 (150, 154)
175 Clone 5	2 (270, 286)	1 (155)	2 (150, 154)
175 Clone 6	2 (270, 286)	1 (155)	2 (150, 154)

a Eight SNP markers from eight different loci were amplified by PCR using chromogenically labelled primers. Only three of the SNP markers are shown.

The number of the bands from each allele (with the size of each band at that locus in parentheses) from NSP PCR analysis gives a ‘DNA fingerprint’. Each cell line has a unique DNA fingerprint, while the parental MDA-MB-175 cell line and each of the six clones have identical DNA fingerprints, as expected.
